# Modeling Progressive Damage and Failure of Single-Lap Thin-Ply-Laminated Composite-Bolted Joint Using LaRC Failure Criterion

**DOI:** 10.3390/ma15228123

**Published:** 2022-11-16

**Authors:** Xiangjiang Wang, Yao Wang, Yundong Ji, Haixiao Hu, Dongfeng Cao, Kaidong Zheng, Hao Liu, Shuxin Li

**Affiliations:** 1 Hubei Key Laboratory of Advanced Materials Mechanics, Wuhan University of Technology, Wuhan 430070, China; 2 State Key Laboratory of Materials Synthesis and Processing, Wuhan University of Technology, Wuhan 430070, China; 3 Foshan Xianhu Laboratory of the Advanced Energy Science and Technology Guangdong Laboratory, Xianhu Hydrogen Valley, Foshan 528200, China; 4 Institute of Advanced Materials and Manufacturing Technology, Wuhan University of Technology, Wuhan 430070, China

**Keywords:** LaRC criterion, comparisons, thin-ply composites, single-lap, in situ effect, fiber kinking

## Abstract

Thin-ply composite failure modes also significantly differ from conventional ply composite failure modes, with the final failure mechanism switching from irregular progressive failure to direct fracture characterized by a uniform fracture with the reduction of the ply thickness. When open holes and bolt joints are involved, thin-ply-laminated composites exhibit more complex stress states, damage evolution, and failure modes. Compared to the experimental study of thin-ply-laminated composite-bolted joints, there are few reports about numerical analysis. In order to understand the damage evolution and failure mechanism of thin-ply-laminated composites jointed by single-lap bolt, a progressive damage model based on three-dimensional (3D) LaRC failure criterion combined with cohesive element is constructed. Through an energy-based damage evolution method, this model can capture some significant mechanical characteristics in thin-ply-laminated structures, such as the in situ effect, delamination inhibition, and fiber compressive kinking failure. The comparisons between the numerical predictions and experimental observations are made to verify the accuracy of the proposed model. It is found that the predicted stress-displacement curves, failure modes, damage morphologies, etc., are consistent with the experimental results, indicating that the presented progressive damage analysis method displays excellent accuracy. The predicted stress at the onset of delamination is 50% higher than that of the conventional thick materials, which is also consistent with experimental results. Moreover, the numerical model provides evidence that the microstructure of thin-ply-laminated composite performs better in uniformity, which is more conducive to inhibiting the intra-layer damage and the expansion of delamination damage between layers. This study on the damage inhibition mechanism of thin-ply provides a potential analytical tool for evaluating damage tolerance and bearing capabilities in thin-ply-laminated composite-bolted joints.

## 1. Introduction

The thin-ply composites are piled by prepregs with a thickness of less than 100 μm/layer. Compared to conventional thick-ply composites, their superior designability and force balance have drawn increasing interests and quickly taken center stage in research. The production of these composites has advanced in recent years, contributed by the advances in spread-tow thin-ply technology, with the application widely extending to many fields [1,2,3], involving unmanned aircraft, high-altitude satellites, and sailing ships. Thin-ply composites are widely anticipated and referred to as “the next generation of high-performance composites”.

The in situ effect lies the most typical mechanical property of thin-ply composites. It means the obvious thickness dependence of transverse tensile strength of composite laminates. In this way, the transverse tensile strength is decreased with the increase of 90° ply thickness until reaching the test strength of the unidirectional laminates. Thin-ply composite failure modes also significantly differ from conventional ply composite failure modes, with the final failure mechanism switching from irregular progressive failure to direct fracture characterized by a uniform fracture with the reduction of the ply thickness [4,5]. Pinho et al. [6] found that the geometric restrictions of the adjacent non-codirectional ply can alter the stress distribution of the key layer, which produces a positive size-strengthening impact, and induces an increase in transverse tensile strength and shear strength. Huang et al. [7,8] pointed out that shear deformation in thin-ply composites is non-uniform, where the maximum shear stress level should be lower than in conventional composites, and the lower shear stress level plays an important role in the higher damage resistance of the thin-ply composites. Gergely et al. [9] pointed out that the shear nonlinearity in the thin-ply composites led to the uniform dispersion of cracks by inhibiting the effect of crack propagation, accompanied by a higher density, which in turn increasingly dissipated the external work. In the tensile test of [+α/–α]_ns_ (α = 0°, 10°, 20°, 30°, 45°, 60°) thin-ply composites, Fuller and Wisnom [10] found that thin-ply laminates have shown that the shear nonlinearity in the thin-ply-laminated composites delays the crack propagation and leads to the uniform dispersion of the cracks and the higher crack density, which dissipates more external work. The thin-ply composite showed significant pseudo-ductility behavior, with a failure strain of up to 6.8% when α = 45°, while the pure 90° thin-ply composite showed no apparent ductility, with a failure strain of only 1.5%. On the one hand, it shows that the reduction of ply thickness can increase the ductility of composite materials and delay the initial damage; on the other hand, it shows again that the strengthening of thin-ply composite materials is not an intrinsic property of the material, but depends on the geometric constraints from adjacent ply and relative angle of the adjacent plies.

The stress state, damage initiation, and evolution patterns of thin-ply composites’ will be extremely complex when applied to bolted connections. Only several experiments on bolted connections of thin-ply composites are reported at present. Based on the mechanical properties test of the perforated plate, Amacher et al. [11] have further examined the alteration in the extrusion strength of single-lap joint bolted joints with varied thicknesses of composites. The outcomes indicated that the extrusion strength of the thin laminates at room temperature increased significantly. The research results of Arteiro et al. [12] also indicates that the reduction of ply thickness enabled a marked improvement in the suppression of delamination and matrix cracking in thin-ply braided composite-bolted joint. Cao et al. [13], depending on the X-ray micro-CT and SEM scanning electron microscopy, have observed the damage mechanism and deformation characteristics of the connection area of the thin laminates at specific loading positions during the tensile process. Resulting from an inhibitory effect of the thin laminates composites on the initial damage crack, the typical failure modes, namely the delamination damage, in the conventional thick laminates composites did not represent, which is widely existing in the extrusion failure plane region and the tensile failure plane region. The findings also demonstrated that the thin-ply composites’ initial damage stress was 50~60% higher than that of the conventional thick layer. The shear fracture, composed of fiber kinking, matrix compression failure, and local material crushing, acted as the primary cause of the progressive extrusion failure mechanism of the thin-ply composite laminates.

It can be concluded from previous studies that thin layering can enhance the transverse strength and shear strength, and the improved nonlinear shear and fracture strain lies the key feature of thin-ply composites [14,15]. Cao et al. [16] proposed the common delamination of standard-ply composite-laminated plates in the bearing failure plane and tensile failure plane was suppressed in thin-ply laminates, resulting in higher bearing failure strength and more damage accumulation based on the experiment of thin-ply-laminated double-lap bolted structure. Wang et al. [17] used SEM to characterize the deformation and damage of the joint under the same number of cycles, and the results showed that the deformation around the hole of thin-laminated plate was about 50% of that around the hole of thick-laminated plate. The extrusion of holes and bolts are required in the working conditions of bolted connection for the thin-ply composites, with their initial failure strength improved. The fiber kinking, matrix compression failure, and local crushing defined by fiber compression develop in the compression zone, diminishing the delamination failure at the edge of the hole that frequently occurs in conventional composites. However, most of the numerical models are unable to accurately predict these failure modes of thin-ply laminates, a numerical model that can accurately predict the failure of thin-layer composites is urgently needed.

Numerical analysis for thin-ply composites is challenging. The factors referring to nonlinear shear, matrix compression failure, fiber kinking, and thin-ply strength require to be considered in the constitutive model of thin-ply composites. The physical processes of thin-ply composites cannot be comprehensively and accurately described by conventional failure criteria, maximum stress, maximum strain, Hoffman, Chang-Chang, Tsai-Wu, and Hashin, and other non-physical phenomenological strength theories [18]. The LaRC criterion [19,20] as an advanced failure criterion was developed based on the Puck failure criterion [21,22], with the in situ effect, nonlinear shear, and fiber kinking effect of laminates considered, covering all the essential properties of thin-ply composites when connected by bolts. Liu et al. [23] first constructed a numerical model based on the LaRC criterion to analyze and discuss the initial failure and stiffness degradation process of the composites under the complex stress state of laminates with holes. Zhang et al. [24] proposed that fiber kinking, matrix failure, and interlayer delamination could all be accurately predicted by a numerical model in an open-cell structure. In this study, the damage failure behavior of bolted thin-ply composites was numerically analyzed based on the LaRC criterion, examining the applicability of the LaRC criterion.

This study conducted a numerical analysis using the explicit user material subroutine (VUMAT) of ABAQUS software on the thin-ply composites with bolted connections based on a three-dimensional progressive damage failure model. The cohesive element is taken to simulate the interlayer failure behavior. The progressive damage evolution of thin-ply composites is studied under bolted connection conditions in combination with the energy-based progressive damage evolution, with the local effect, nonlinear shear, matrix compression failure, and fiber compression kinking of thin-ply composites taken into consideration. The numerical method of LaRC initial failure and progressive damage was validated by the experiments of Ref [13]. The numerical results show a suitable agreement with experimental results in failure modes and the damage initiation effect of delamination. The present study is therefore structured in two parts. The first one focuses on the study of the construction of the numerical model, including approaches to implement in situ effect, intralaminar damage initiation, and intralaminar progressive damage. The second one exhibited predicted results of single-lap structure involving stress-displacement curves and progressive damage, and the effectiveness and accuracy of the present numerical model are discussed.

## 2. Numerical Model

### 2.1. Finite Element Models

The accurate prediction of the damage initiation and progressive damage process of fiber is essential for numerical prediction and damage control of the single-lap joint structure of the composites, matrix, and interlayer. On this foundation, a numerical analytic approach to precisely anticipate the single-lap joint structure progressive failure is eventually obtained after examining the matrix damage, fiber damage, and interlayer delamination damage in the single-lap joint structure.

**Figure 1:**
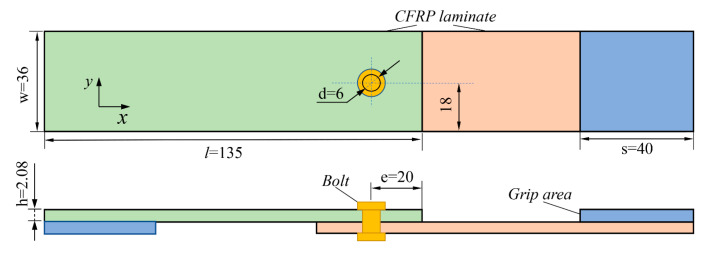
Single-lap joint configuration of T700/2510 CFRP (Unit: mm).

Cao et al. [13] used the carbon fiber-reinforced polymer (CFRP) thin-ply materials T700/2510 to fabricate the composite laminates, with the bolt made of TC21 titanium alloy material. The length of the laminated board is 135 mm, with its thickness of 2.08 mm, and the ply order of [45/0/–45/90]_4S_, where the width-diameter ratio is 6. A reinforcing sheet with a length of 40 mm and a thickness equal to that of the laminated board was used in the clamping area of the specimen to prevent bending deformation of the laminated board during clamping. Figure 1 depicts the specimen’s physical dimensions.

**Figure 2:**
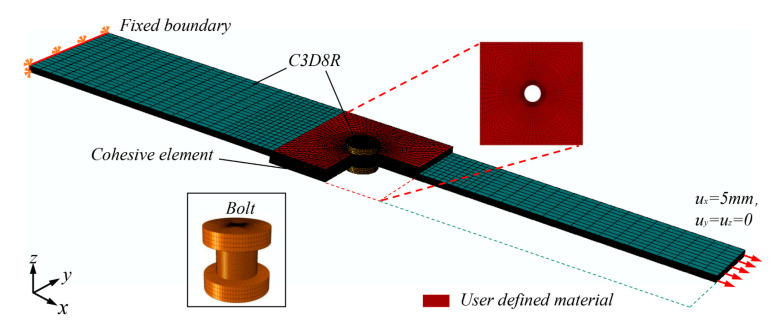
CFRP single-lap finite element model.

Herein, the finite element software ABAQUS/Explicit is applied to establish the dynamics model. The single-lap thickness is 2.08 mm. The boundary conditions fully constrained on the left, and the displacement load of 5 mm is applied along the x direction on the right. Figure 2 shows the three-dimensional finite element model of CFRP single-lap.

**Figure 3:**
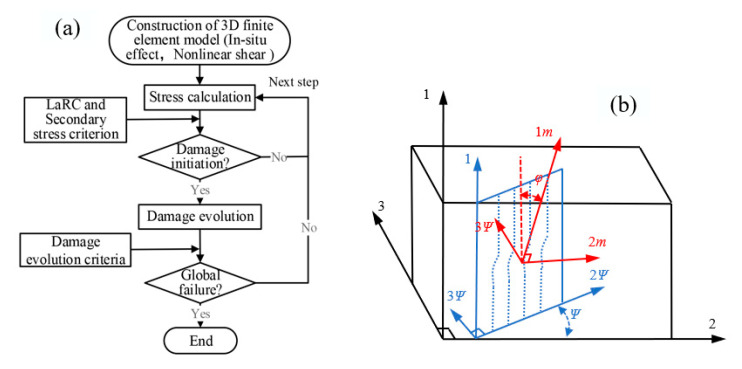
(**a**) Flow chart of numerical calculation; (**b**) fiber kinking coordinate conversion diagram.

The element type of the CFRP material is eight-node 3D solid elements (C3D8R), and the number is 189,440. To prevent the failure of matrix and fiber caused by stress concentration at the hole edge, mesh refinement is carried out in the area around the hole. In the laminate lap area marked red in Figure 2, a total of 19,306 zero-thickness cohesive elements (COH3D8) are inserted. The zero-thickness cohesive elements are inserted into each interface between different plies using a python user-defined code. The bolt element is C3D8R, and the number of elements is 54,400. The area around the hole of the laminate is assigned with the attribute of User Material Subroutines. The initiation and development of intralaminar damage are characterized by the VUMAT subroutine. The mechanical properties of T700/2510 CFRP are listed in Table 1. The technical flow chart is shown in Figure 3a.

### 2.2. In Situ Effect

Camanho et al. [21] proposed the analytical solutions to in situ strength values of laminates with different layering thicknesses and stacking modes from the perspective of fracture mechanics. The equation of in situ effect is shown in Table 2. In the numerical calculation, the tensile strength and shear strength enhancement of the material were calculated in Table 2.

Where $Y_{T}^{}$ and $S_{L}^{}$ represent the transverse tensile and shear strength, respectively; $Y_{T}^{\mathrm{is}}$ and $S_{L}^{\mathrm{is}}$ denote the in situ transverse tensile and shear strength, respectively; β indicates the nonlinear factor; $G_{I}^{C}$ and $G_{II}^{C}$ refer to the I and II interlaminar fracture energy, respectively; $\Lambda_{22}^{0}=2\left(1/E_{22}-v_{21}^{2}/E_{11}\right)$, $E_{11}$, and $E_{22}$ represent Young’s modulus; $v_{21}$ indicates the Poisson ratio; t stands for the thickness of the UD ply.

### 2.3. Intralaminar Damage Initiation

The initiation of damage in CFRP materials is assessed against the LaRC initial failure. The LaRC criterion is detailed as follows:

(1) Fiber tension criterion

The maximum stress in material coordinate system is taken as the start of tensile failure, when the maximum longitudinal stress reaches the ultimate tensile strength, as shown in Equation (1).

(1) $$f_{ft}=\frac{\sigma_{11}}{X_{T}}=1,\;\sigma_{11}>0$$

where stress state is computed in the global coordinate system; $\sigma_{11}$ indicates the tensile stress in the longitude direction; $X_{T}$ denotes the longitude tensile strength of unidirectional laminates; $f_{ft}$ refers to the fiber tensile failure factor. When $f_{ft}\geq 1$, the tensile damage caused to the fiber is initiated.

(2) Fiber kinking damage

Fiber kinking results from the defects in local fiber or the flaws in processing, such as fiber deflection or splitting. Given the initial fiber dislocation in the composite material, the shear stress between the misaligned fibers exacerbates fiber deflection, which further increases the shear stress until failure of the fiber. Pinho et al. [26] introduced the characteristics of three-dimensional fiber kinking failure into the two-dimensional kinking model, the model coordinate diagram of which is shown in Figure 3b.

The $1-2^{}-3^{}$ represents the material coordinate system; ψ indicates the angle between the kinking plane and axis 2; φ refers to the deflection angle of coordinate systems $1-2^{\psi }-3^{\psi }$ and $1^{m}-2^{m}-3^{\psi }$. Fiber kinking occurs in the plane of local frame $1^{m}-2^{m}-3^{\psi }$. The transformation formula of stress in the above coordinate system is expressed as Equations (2) and (3).

(2) $$\left\{\begin{array}{l} \sigma_{22}^{\psi }=\sigma_{22}\cos^{2}\psi +\sigma_{33}\sin^{2}\psi +2\tau_{23}\sin\psi \cos\psi \\ \tau_{12}^{\psi }=\tau_{12}\cos\psi +\tau_{31}\sin\psi \\ \tau_{23}^{\psi }=\left(\sigma_{33}-\sigma_{22}\right)\sin\psi \cos\psi +\tau_{23}\left(\cos^{2}\psi -\sin^{2}\psi \right)\\ \tau_{31}^{\psi }=\tau_{31}\cos\psi -\tau_{12}\sin\psi \end{array}\right.$$

(3) $$\left\{\begin{array}{l} \sigma_{22}^{m}=\sigma_{11}\sin^{2}\varphi +\sigma_{22}\cos^{2}\varphi -2\tau_{12}^{\psi }\sin\varphi \cos\varphi \\ \tau_{12}^{m}=\left(\sigma_{22}^{\psi }-\sigma_{11}\right)\sin\varphi \cos\varphi +\tau_{12}^{\psi }\left(\cos^{2}\varphi -\sin^{2}\varphi \right)\\ \tau_{23}^{m}=\tau_{23}^{\psi }\cos\varphi -\tau_{31}^{\psi }\sin\varphi \end{array}\right.$$

where $\sigma_{22},\sigma_{33},\tau_{23},\tau_{12}$ and $\tau_{31}$ represent the stresses in the material coordinate system. When $\sigma_{11}\leq 0$, the criterion of fiber kinking damage initiation based on the fiber kinking coordinate system can be expressed as:

(4) $$f_{\mathrm{kink}}={\left(\frac{\tau_{23}^{m}}{S_{T}-\mu_{T}\sigma_{22}^{m}}\right)}^{2}+{\left(\frac{\tau_{12}^{m}}{S_{L}^{\mathrm{is}}-\mu_{L}\sigma_{22}^{m}}\right)}^{2}+{\left(\frac{\langle \sigma_{22}^{m}\rangle }{Y_{T}^{\mathrm{is}}}\right)}^{2}=1$$

where $\langle \cdot \rangle$ represents Macauley bracket, $\langle x\rangle =\left(x+\left|x\right|\right)/2$; $S_{T}^{}$ indicates the transverse shear strength; $S_{L}^{\mathrm{is}}$ denotes the longitudinal shear in situ strength; $Y_{T}^{\mathrm{is}}$ refers to the transverse tensile in situ strength; $\sigma_{n}$ means the normal stress on the fracture surface; $\mu_{T}$ and $\mu_{L}$ represent friction coefficients; $f_{\mathrm{kink}}$ denotes the fiber kinking failure factor. When $f_{\mathrm{kink}}\geq 1$, it suggests the initiation of fiber damage.

(3) Nonlinear shear

**Figure 4:**
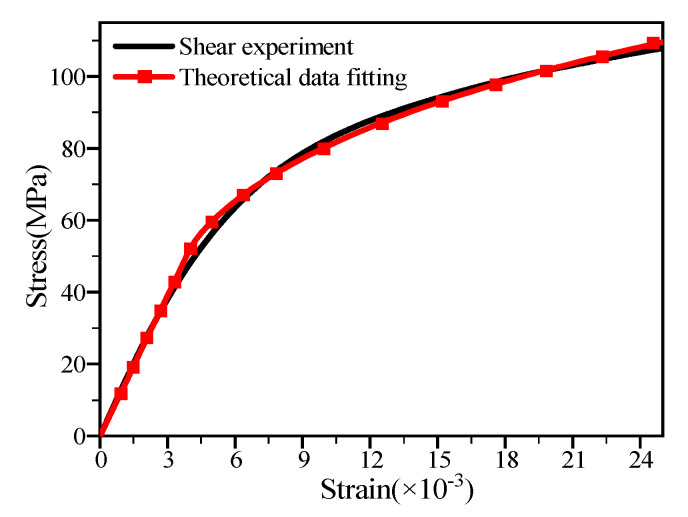
T700 CFRP material shear nonlinearity fitting curve.

Due to the various ways stress is applied in the pore area of the single-lap structure of thin-laminated composite and the nonlinearity of shear force acting on the composite, the pore area of the structure will show obvious nonlinearity when loaded. Herein, the Ramberg–Osgood model is adopted to characterize the nonlinearity of shear force acting on composite laminates [27]. The nonlinearity between shear stress and shear strain is expressed as follows:

(5) $$\tau =\frac{G\gamma }{{\left[1+{\left(\frac{G\gamma }{\tau_{0}}\right)}^{n}\right]}^{1/n}}$$

where G, γ, and $\tau_{0}$ represent shear modulus, engineering shear strain, and ultimate shear stress, respectively; n indicates a nonlinear factor, which is fitted through in-plane shear experiment data of T700 CFRP laminates. Figure 4 shows the fitting curve of in-plane shear test results and numerical calculation for T700 CFRP material. The best-fitted curve with experimental data gives the value of factor ‘n’ is equal to 0.9, hence is used for the following numerical simulation.

In general, the matrix compression sample is damaged by shearing [22]. As revealed by the experimental test, the angle between the failure surface and the thickness direction of the composite sample is about 53° [28]. The difference from the theoretical value is attributable to the friction occurring on the composite surface. For each included angle on the fracture surface, the direction of the fracture surface depends on a particular combination of shear stress ($\tau_{T}$ and $\tau_{L}$) and normal stress ($\sigma_{n}$). The stress component of the fracture surface under a 3D stress state can be expressed as:

(6) $$\begin{array}{l} \sigma_{n}\left(\phi \right)=\sigma_{22}\cos^{2}\phi +\sigma_{33}\sin^{2}\phi +2\tau_{23}\sin\phi \cos\phi \\ \tau_{T}\left(\phi \right)=-\sigma_{22}\sin\phi \cos\phi +\sigma_{33}\sin\phi \cos\phi +\tau_{23}\left(\cos^{2}\phi -\sin^{2}\phi \right)\\ \tau_{L}\left(\phi \right)=\tau_{31}\sin\phi +\tau_{21}\cos\phi \end{array}$$

(4) Matrix tensile failure

The longitudinal tensile and transverse tensile in situ strength are considered for matrix tensile failure. The angle of fracture surface is determined by conducting search through the traversal method. The intensity factor of matrix tensile failure stress is expressed as Equation (7), where $\tau_{T}$ represents the transverse shear stress; $\tau_{L}$ refers to the transverse normal stress. When $f_{mt}\geq 1$, it indicates the initiation of matrix tensile damage.

(7) $$f_{\mathrm{mt}}={\left(\frac{\tau_{T}}{S_{T}-\mu_{T}\sigma_{n}}\right)}^{2}+{\left(\frac{\tau_{L}}{S_{L}^{\mathrm{is}}-\mu_{L}\sigma_{n}}\right)}^{2}+{\left(\frac{\sigma_{n}}{Y_{T}^{\mathrm{is}}}\right)}^{2}=1,\;\sigma_{n}\geq 0$$

(5) Matrix compression failure

Therefore, the stress intensity factor $f_{mc}$ of matrix compression failure occurring on the fracture surface can be expressed as Equation (8). When $f_{mc}\geq 1$, it indicates the initiation of damage induced by matrix compression failure.

(8) $$f_{\mathrm{mc}}={\left(\frac{\tau_{T}}{S_{T}-\mu_{T}\sigma_{n}}\right)}^{2}+{\left(\frac{\tau_{L}}{S_{L}^{\mathrm{is}}-\mu_{L}\sigma_{n}}\right)}^{2}=1,\;\sigma_{n}<0$$

### 2.4. Intralaminar Progressive Damage Model

The energy-based linear constitutive is adopted to describe the evolution behavior of materials after the initiation of damage. In the damage evolution stage, the damage state as a variable is introduced to characterize the degree of degradation shown by the materials after failure. For each failure mode, damage state d=0 represents the initial time at which damage occurs, while d=1 represents the moment at which complete failure occurs. The value of the damage state variable can be defined as:

(9) $$d=\max\left\{0,\min\left\{1,\varepsilon_{\mathrm{eq}}^{f}\frac{\varepsilon_{\mathrm{eq}}-\varepsilon_{\mathrm{eq}}^{0}}{\varepsilon_{\mathrm{eq}}\left(\varepsilon_{\mathrm{eq}}^{f}-\varepsilon_{\mathrm{eq}}^{0}\right)}\right\}\right\}$$

where $\varepsilon_{\mathrm{eq}}$, $\varepsilon_{\mathrm{eq}}^{0}$, and $\varepsilon_{\mathrm{eq}}^{f}$ represent the equivalent strains at the present time, the onset of failure, and the time of complete failure, respectively. The factors driving the development of damage caused to fiber and matrix under tensile load differ from those under compressive load. The damage evolution methods under different damage modes are proposed according to different damage initiation criteria.

(1) Damage evolution criteria for tensile and compressive failure of the matrix

In the event of matrix failure, the equivalent stress and strain of the section are expressed as:

(10) $$\sigma_{\mathrm{eq},I}=\sqrt{{\langle \sigma_{n}\rangle }^{2}+{\left(\tau_{T}\right)}^{2}+{\left(\tau_{L}\right)}^{2}}$$

(11) $$\varepsilon_{\mathrm{eq},I}=\frac{\left|\sigma_{n}\varepsilon_{n}\right|+\left|\tau_{T}\gamma_{T}\right|+\left|\tau_{L}\gamma_{L}\right|}{\sigma_{\mathrm{eq},I}}$$

where I is used to distinguish between two failure modes: matrix stretching ($\sigma_{n}\geq 0$) and matrix compression ($\sigma_{n}\leq 0$); $\varepsilon_{n}$ denotes the normal strain on the fracture surface; $\gamma_{T}$ and $\gamma_{L}$ refer to the tangential strains on the fracture plane. When the matrix completely fails, the equivalent strain can be expressed as:

(12) $$\varepsilon_{\mathrm{eq},I}^{f}=\frac{2G_{m}}{\sigma_{\mathrm{eq}}^{0}L}$$

where *L* represents the characteristic length of the element; $G_{m}$ indicates the fracture energy generated when the matrix fails; $\sigma_{\mathrm{eq}}^{0}$ denotes the equivalent stress at the time of damage initiation on the section. The fracture energy can be determined by applying the hybrid fracture criteria, such as the power-law criterion:

(13) $$G_{m}=G_{\mathrm{IC}}^{m}{\langle \frac{\sigma_{n}^{0}}{\sigma_{\mathrm{eq},I}^{0}}\rangle }^{2}+G_{\mathrm{IIC}}^{m}{\left(\frac{\tau_{T}^{0}}{\sigma_{\mathrm{eq},I}^{0}}\right)}^{2}+G_{\mathrm{IIC}}^{m}{\left(\frac{\tau_{L}^{0}}{\sigma_{\mathrm{eq},I}^{0}}\right)}^{2}$$

where $G_{\mathrm{IC}}^{m}$ and $G_{\mathrm{IIC}}^{m}$ represent the in-plane type I and type II fracture energies (interlayer fracture energy is used in this study) of the matrix, respectively; $\sigma_{n}^{0}$ indicates the normal stress on the fracture surface at the time when failure is initiated; and, similarly, $\tau_{T}^{0}$ and $\tau_{L}^{0}$ refer to the tangential stress components on the fracture plane at the time when failure is initiated.

(2) Damage evolution criterion of fiber tensile and kinking failure

In the case of fiber tensile failure, damage evolution occurs, which depends on the strain state in the material coordinate system. The $\varepsilon_{\mathrm{eq},f}^{f}$ equivalent strain is expressed as:

(14) $$\varepsilon_{\mathrm{eq},f}^{f}=\frac{2G_{f}}{\sigma_{\mathrm{ft}}^{0}L}$$

where $G_{f}$ represents the fracture energy of fiber tensile failure; $\sigma_{\mathrm{ft}}^{0}$ indicates the stress in the direction of fiber at the time when damage is initiated in the material coordinate system. The process of kinking failure that occurs to the fiber is related to the deflection of the fiber kinking band. In addition, this process can be controlled by the shear stress $\sigma_{\mathrm{kink}}=\tau_{12}^{m}$. The equivalent strain that leads to the development of fiber damage is expressed as $\varepsilon_{\mathrm{kink}}=\gamma_{12}^{m}$. The corresponding transformation formula is presented as follows:

(15) $$\begin{array}{l} \varepsilon_{11}^{\psi }=\varepsilon_{11}\\ \varepsilon_{22}^{\psi }=\varepsilon_{22}\cos^{2}\psi +\varepsilon_{33}^{}\sin^{2}\psi +\gamma_{23}\sin\left(\psi \right)\cos\left(\psi \right)\\ \gamma_{12}^{\psi }=\gamma_{12}\cos\left(\psi \right)+\gamma_{13}\sin\left(\psi \right)\\ \gamma_{12}^{m}=-\varepsilon_{11}^{\psi }\sin(2\varphi )+\varepsilon_{22}^{\psi }\sin(2\varphi )+\gamma_{12}^{\psi }\cos(2\varphi ) \end{array}$$

The fiber kinking failure strain is expressed as:

(16) $$\varepsilon_{\mathrm{kink}}^{f}=\frac{2G_{\mathrm{kink}}}{\sigma_{\mathrm{kink}}^{0}L}$$

where $G_{\mathrm{kink}}$ represents the kinking fracture energy of fiber; $\sigma_{\mathrm{kink}}^{0}$ indicates the equivalent stress at the time when $f_{\mathrm{kink}}=1$; *L* denotes the characteristic length of the element.

(3) Fiber and matrix degradation

The damage evolution of single-lap structure fiber and matrix is explored by introducing the damage factors $d_{i}(i=\mathrm{ft},\mathrm{fc},\mathrm{mt},\mathrm{mc})$ to characterize the extent of degradation in performance after failure. The damage factors $d_{i}$ are substituted into the stiffness matrix, and the structural stiffness is reduced by lowering Young’s modulus of the material. The reduction of stiffness is expressed as follows:

(17) $$\begin{array}{l} E_{11}^{d}=\left(1-d_{f}\right)E_{11},\;E_{22}^{d}=\left(1-d_{f}\right)\left(1-d_{m}\right)E_{22},\\ E_{33}^{d}=\left(1-d_{f}\right)\left(1-d_{m}\right)E_{33},\\ G_{i}^{d}=\left(1-d_{g}\right)G_{i}^{s}\;(i=12,13,23),\\ d_{f}=1-\left(1-d_{\mathrm{ft}}\right)\left(1-d_{\mathrm{fc}}\right),\;d_{m}=\max\left(d_{\mathrm{mt}},d_{\mathrm{mc}}\right),\\ d_{g}=1-\left(1-d_{\mathrm{ft}}\right)\left(1-d_{\mathrm{fc}}\right)\left(1-S_{\mathrm{mt}}d_{\mathrm{mt}}\right)\left(1-S_{\mathrm{mc}}d_{\mathrm{mc}}\right) \end{array}$$

where $S_{\mathrm{mt}}$ and $S_{\mathrm{mc}}$ represent the correction coefficients required to restrict the impact of shear modulus loss. Given the constraint of bolts on the further development of damage around the hole of laminate in the single-lap structure of thin-ply composite material, the value of $S_{\mathrm{mt}}$ and $S_{\mathrm{mc}}$ is set to 0.7 and 0.2 [24] in the numerical calculation model used in this study, respectively.

### 2.5. Interlaminar Damage Model

Cohesive elements are used to simulate interlaminar delamination in the thin-ply composite. The zero-thickness cohesive elements are inserted into each interface between different plies using a python user-defined code. Cao et al. used cohesive elements to well predict laminate delamination [28,29,30,31]. Especially the damage initiation and evolution method of bilinear constitutive is widely used to address the layered damage caused to composite laminates. The tract-separation criterion that is applicable under the bilinear mixing mode is adopted to predict the delamination damage occurring to the composite laminate single-lap structure under the secondary bending effect. The effective displacement in the mixed mode can be expressed as Equation (18), and the state variables in the damage evolution process can be expressed as Equation (19):

(18) $$\delta_{m}=\sqrt{{\langle \delta_{1}\rangle }^{2}+\delta_{2}{}^{2}+\delta_{3}{}^{2}}$$

(19) $$d=\frac{\delta_{m}^{f}(\delta_{m}^{\max}-\delta_{m}^{0})}{\delta_{m}^{\max}(\delta_{m}^{f}-\delta_{m}^{0})}\;(d\in [0,1])$$

where $\delta_{1}$, $\delta_{2}$, and $\delta_{3}$ represent the effective displacement components of the interface in three directions, respectively; $\delta_{m}^{0}$ and $\delta_{m}^{f}$ denote the effective displacements of the interface at the time of damage initiation and complete failure, respectively; $\delta_{m}^{\max}$ refers to the maximum relative displacement of the interface. In this study, the criterion of secondary stress damage initiation and that of damage evolution based on POWER LOW energy are adopted. They are expressed as follows:

(20) $${\left(\frac{\langle t_{1}\rangle }{N}\right)}^{2}+{\left(\frac{t_{2}}{S}\right)}^{2}+{\left(\frac{t_{3}}{T}\right)}^{2}=1$$

(21) $${\left(\frac{G_{I}}{G_{\mathrm{IC}}}\right)}^{2}+{\left(\frac{G_{\mathrm{II}}}{G_{\mathrm{IIC}}}\right)}^{2}+{\left(\frac{G_{\mathrm{III}}}{G_{\mathrm{IIIC}}}\right)}^{2}=1$$

where $t_{1}$, $t_{2}$, and $t_{3}$ refer to the normal stresses and shear stresses in two directions, respectively; N, S, and T indicate the normal strength and shear strength in two directions, respectively. $G_{I}$, $G_{\mathrm{II}}$, and $G_{\mathrm{III}}$ denote the energy release rate in the current state; $G_{\mathrm{IC}}$, $G_{\mathrm{IIC}}$, and $G_{\mathrm{IIIC}}$ represent the critical fracture energy corresponding to the three failure modes of the interface, respectively.

## 3. Numerical Results and Discussion

### 3.1. Stress-Displacement Curve of Single-Lap Structure

**Figure 5:**
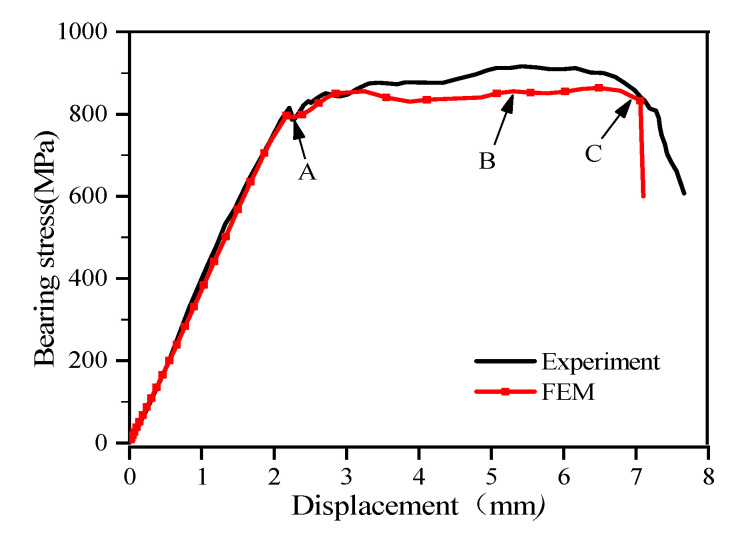
Stress-displacement curves of thin-layer composite material single-lap structures.

The stress-displacement curve as extracted from the single-lap structure of the thin-ply composite is shown in Figure 5, where the stress-displacement curves experiment [13] and finite element modeling (FEM) indicate the experimental results and numerical calculation results, respectively. As shown in Figure 5, the change of displacement and load between the initial loading and the overall failure of the structure can be determined. As for the extrusion stress of the laminated composite single-lap, it is defined as the tensile load divided by the product of the diameter of the connecting hole and the thickness of the laminated composite single-lap. Specifically, the equivalent extrusion stress is calculated by using:

(22) $$\sigma_{\mathrm{bearing}}=\frac{k\times P}{d\times h}$$

where P represents the tensile load in the loading process; k indicates the load coefficient of the connection hole, which is equal to the number of bolted joints (single-lap is 1.0); d and h refer to the diameter of the connecting hole and the thickness of the laminate, respectively.

Figure 5 gives stress-displacement curves of single-lap thin-ply-laminated composite-bolted joint, and corresponding experimental data are also shown for comparison. In general, the experimental results [13] are well consistent with the predicted results by FEM. In Figure 5, states A, B, and C indicate the initiation of the “pseudoplastic phase”, peak bearing capacity, and overall failure state of the single-lap structure of the thin-ply composite, respectively. The peak bearing stress of the experiment and FEM were 910 MPa and 865 MPa, separately. The stress level of numerical prediction is below that of corresponding experimental results between states B and C, while the deviations are not small. These deviations mainly attribute to the inaccuracy of the stiffness/strength degradation factor in the intralaminar progressive damage model (Section 2.4). In this work, the damage state variable, d, is defined by a linear degradation, as shown in Equation (9). This simple definition does not accurately reflect the complex coupling effects of various failure modes, which is a worldwide tough issue in the composite simulation field.

**Figure 6:**
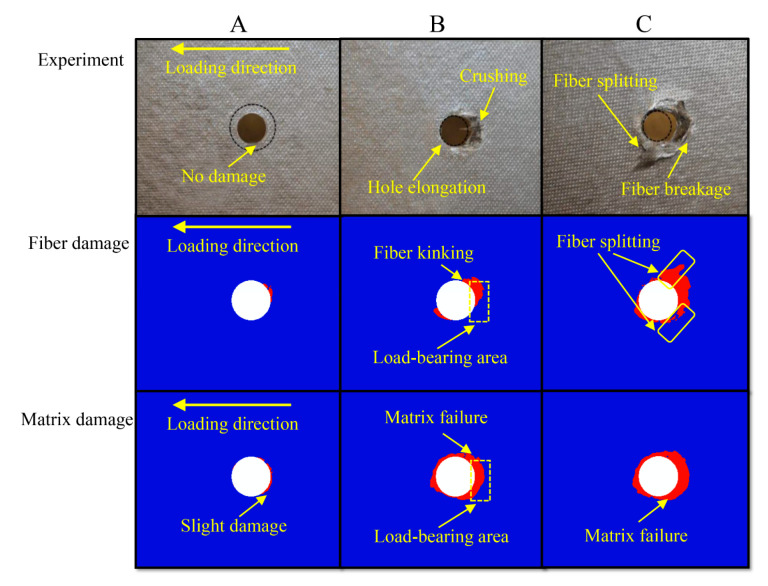
Experimental results [13] and numerical calculation of damage states around holes at states A, B, and C on the lower surface.

The whole process is divided into two major stages. One is the loading stage (before state A in Figure 5), which is dominated by elastic deformation before the stiffness drops slightly. The other is the period during which the composite materials demonstrate similar structural properties to the metallic materials showing yield deformation (state A–C in Figure 5). At this stage, the extrusion stress in laminated sheet rises nonlinearity as the displacement increases, which is due to the cumulative damage caused around the hole of the composite material (in Figure 6). The fiber-matrix splitting is due to the tensile bending stresses arising from transverse loading. In this stage, the stiffness of bolted joint drops considerably and the load-displacement curve exhibits significant nonlinear behavior [13]. A further increase will cause the load bolt to reach around the hole of the damaged laminate, and the thin-ply composite performs well in reducing the damage, which leads to a nonlinear increase at a slow pace for loading in the load stress and displacement curve, rather than a sharp decline.

### 3.2. Progressive Damage State of Single-Lap Structure

To explore the accumulation of damage of the single-lap structure of thin-laminated composite under different conditions, the states of experimental and numerical damage around the hole of the laminate at states A, B, and C in Figure 5 were extracted, respectively.

When the single-lap structure is loaded, the bolt deflects due to the secondary bending effect, which leads to the uneven distribution of extrusion action of the screw around the hole. Due to the significant extrusion effect of the screw on the load-bearing area lower surface of the upper lap plate, the load-bearing area of the lower surface is damaged first. Due to the consistency of the stress state and damage mechanism between the upper lap plate and the lower lap plate, as well as the concentration of damage caused to the upper lap plate in the area that is around the hole and close to the lower surface, the damage state of the lower surface around the hole of the upper lap plate (corresponding to the surface layer in Figure 6) as shown by the results of experimental [13] and numerical calculation is extracted for analysis. The damage state around the pore is shown in Figure 6, where the damage state of the numerical calculation results can be divided into fiber damage state and matrix damage state (the failure of the element is marked red in the figure).

The main forms taken by damage caused to the composite single-lap structures under loading are fiber kinking failure, matrix compression failure and the interlayer delamination damage caused by the failure of cohesive elements. As shown in Figure 6, when the structure is loaded to state A, there is no visible damage observed in the edge regions beneath the laminate. According to the numerical calculation results shown in Figure 6, the bolt deflects to a certain extent because of the secondary bending effect, which enhances the extrusion effect exerted in the middle part of the screw on the laminate, thus causing fiber kinking failure at the state of contact between the lower surface of the laminate and the middle part of the screw. In this case, the matrix near the fiber reaches the failure condition only rarely reaches the failure condition, and the bolt imposes a strong out-of-plane constraint on the laminate, which suppresses fiber kinking failure. Therefore, a tiny part of damage around the pore cannot be directly observed from the experimental results. With the gradual accumulation of damage and deformation around the hole when the structure is loaded to state A (especially after state B), the damage around the hole develops slowly to the extent that it is clearly observable. In this case, the experimental results demonstrate clearly that bolt extrusion damage and hole elongation occur in the edge regions beneath the laminate (Figure 6).

According to Figure 5 and Figure 6, the stress-displacement curve increases linearly before the structure is loaded to state A. After the loading to state A, the load-bearing capacity of the structure is reduced as the damage and deformation accumulate around the hole. Meanwhile, the damage is inhibition by the thin-laminated composite, which delays the further development of damage caused to the structure. Therefore, the stress-displacement curve beyond state A shows a nonlinear increase under the combined effect of the damage accumulation around the hole, the damage initiation effect of the thin-layer material, and the nonlinear shear of the material. When the loading reaches state B, damage occurs to much of the fiber and matrix around the hole of the laminate. Due to the non-uniform distribution of bolt extrusion caused by the secondary bending effect, screw ingression and hole elongation occur on the right around the hole of the laminate. When the structure is loaded to state C, the whole screw enters the laminate. At this time, the damage caused to the fiber around the hole of the laminate expands along the direction of 45°, which leads to splitting damage. In addition, the damage caused to the matrix can be observed on the right side of the hole with no direct contact with the screw. Due to the extensive fiber splitting and matrix damage around the hole, the structure of the laminate loses its load-bearing capacity.

**Figure 7:**
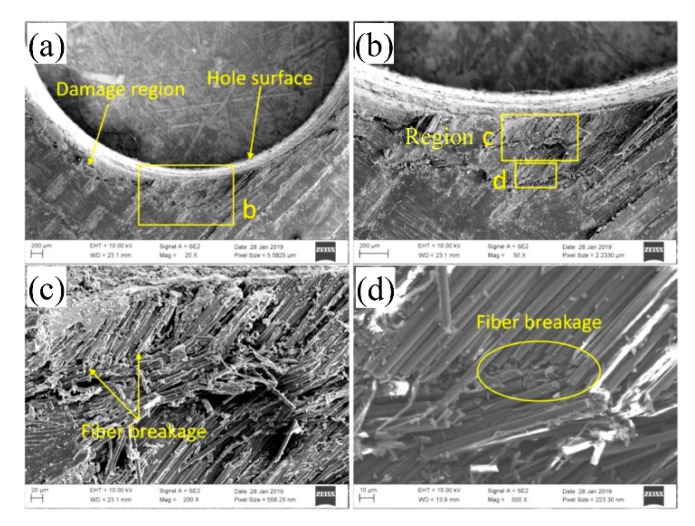
SEM scanning results of hole circumference at A [13].

**Figure 8:**
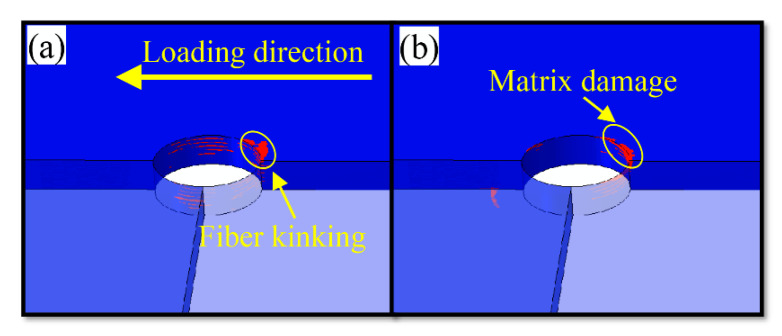
FEM results of hole circumference at A ((**a**) fiber kinking, (**b**) matrix damage).

The SEM [13] results are shown in Figure 7. On this basis, the numerical calculation results obtained for state A were selected for comparative analysis. In addition, the damage state in the three-dimensional area around the hole on the lower surface of the upper lap plate is selected, as shown in Figure 8.

According to the results of SEM scanning in Figure 7 and those of numerical simulation in Figure 8, both the fiber and the matrix in the pore area of the laminate suffer damage. As shown in Figure 8, the extrusion damage caused to the screw around the hole concentrates in the area close to the upper surface (the lower surface of the upper lap plate), while the damage caused to the lower surface concentrates in the area close to the left edge of the nut. This is because the secondary bending effect leads to the deflection of the bolt, which enhances the extrusion effect of the middle part of the screw on the hole and the shear effect of the nut edge on the laminate surface. The increase in localized stress leads to premature damage of the laminate in the stress riser, which accelerates the development of damage in the later phase.

**Figure 9:**
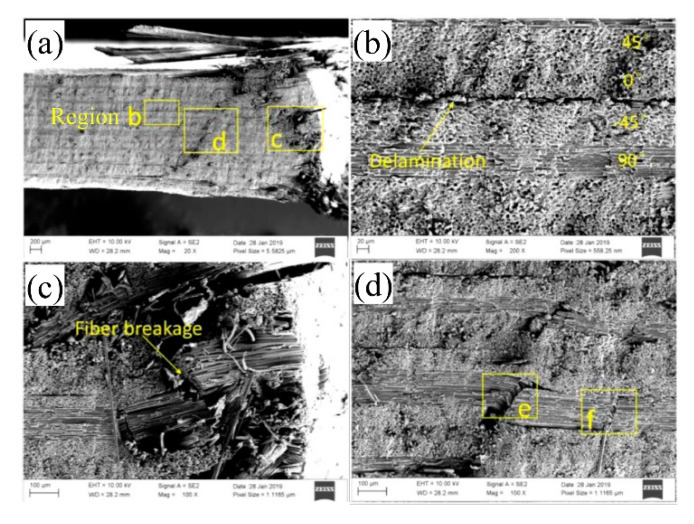
Scanning results of SEM around the hole at B [13] ((**a**–**d**) region).

**Figure 10:**
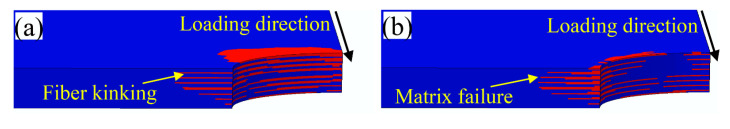
FEM results of circumferential section at B ((**a**) fiber kinking failure, (**b**) matrix damage).

The results of SEM and numerical calculation in the B state were compared and analyzed. Since the single-lap structure is symmetric, the scanning area is set to be half the cross-section along the direction of vertical loading around the hole. Figure 9 and Figure 10 show the SEM scanning results and numerical calculation results obtained for half cross-section in the direction of vertical loading around the hole are shown in, respectively.

As shown in Figure 9 and Figure 10, fiber kinking failure and matrix damage commonly occur in the cross-section along the direction of vertical loading around the hole at state B. The distribution of damage resembles a wedge. According to Figure 10, which shows fiber kinking failure, except for the fiber kinking failure that occurs in the 90° layer along the direction of vertical loading around the hole, no other layers suffer fiber kinking damage. However, the fiber kinking failure occurring in the 90° cross-section along the direction of vertical loading around the hole exacerbates the damage of interlayer delamination between adjacent layers, which restricts the obvious delamination damage to the 90° fiber layer of the thin-ply composite. Delamination damage is not commonly observed in the conventional thickness laminate during the extrusion.

Figure 10 shows that the range of damage caused by screw extrusion is gradually reduced from the upper surface to the lower surface, suggesting that the secondary bending effect leads to the uneven distribution of extrusion stress in the area around the hole of the screw and laminate. Furthermore, the uneven distribution of stress causes fiber kinking failure and matrix damage to occur early in some areas around the pore. In addition, the secondary bending effect contributes to enhancing the contact effect between the left side of the nut and the laminate, which diminishes the contact effect between the right side and the laminate. This increases and decreases the normal constraints on the left and right around the hole of the upper lap plate, respectively. However, the reduction in the right normal constraint causes the extrusion deformation on the right to expand out of the plane, which plays an important role in the fiber kinking and interlayer delamination of the upper lap plate on the right.

**Figure 11:**
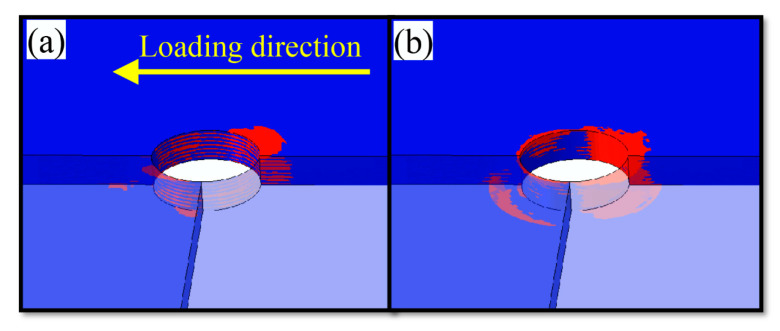
FEM results of hole circumference at B ((**a**) fiber kinking, (**b**) matrix damage).

**Figure 12:**
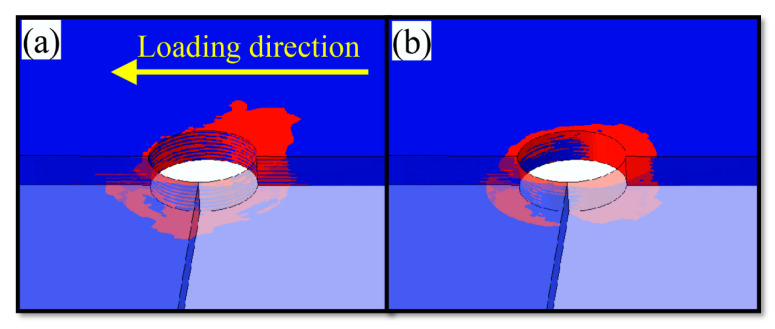
FEM results of hole circumference at C ((**a**) fiber kinking, (**b**) matrix damage).

To study the evolutionary trend of damage between state B and state C, the damage state around the hole of the laminate is extracted from the numerical calculation results obtained at state B and state C, respectively. The extracted results are shown in Figure 11 and Figure 12. Upon comparison between the two figures, it can be found that more damage occurs in contact between the hole circumference and the screw of the laminate at state B and state C and that the fiber kinking failure along the 45° direction at state C is significantly more evident than at state B. According to Figure 12b, the range of damage caused to the matrix around the hole of the laminate at state C has also expanded into the ±45° region around the hole. Fiber kinking failure occurs commonly in the ±45° area around the pore at state C, and more severe matrix damage occurs in this area, which causes fiber kinking failure to progress into fiber splitting damage. When the severity of fiber splitting and matrix damage around the hole reaches a certain level, the laminate will cease to withstand the ingression of the screw and reach the bearing limit of the structure. In addition, the progressive damage to the structure is also exacerbated by the reduction in surface normal constraint caused by the secondary bending effect and the non-uniform extrusion of the screw.

### 3.3. Analysis of Progressive Failure Mechanism of Single-Lap Structure

**Figure 13:**
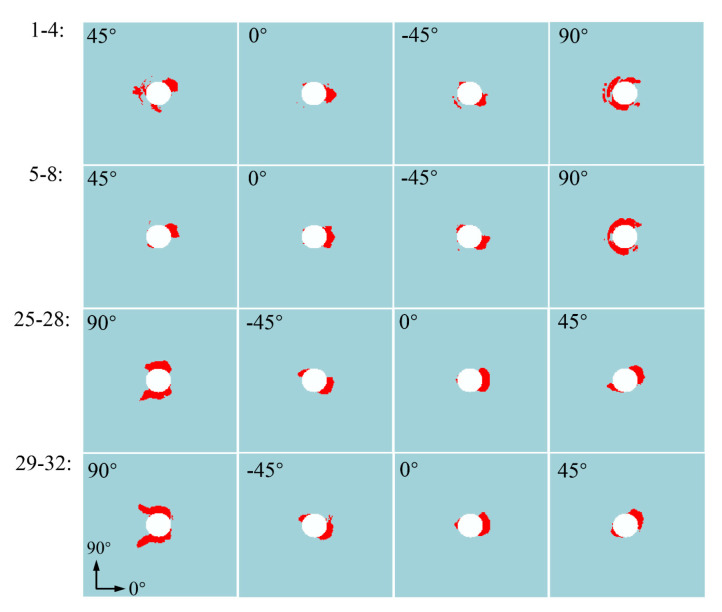
Thin-ply-laminated fiber kinking failure state at point B.

**Figure 14:**
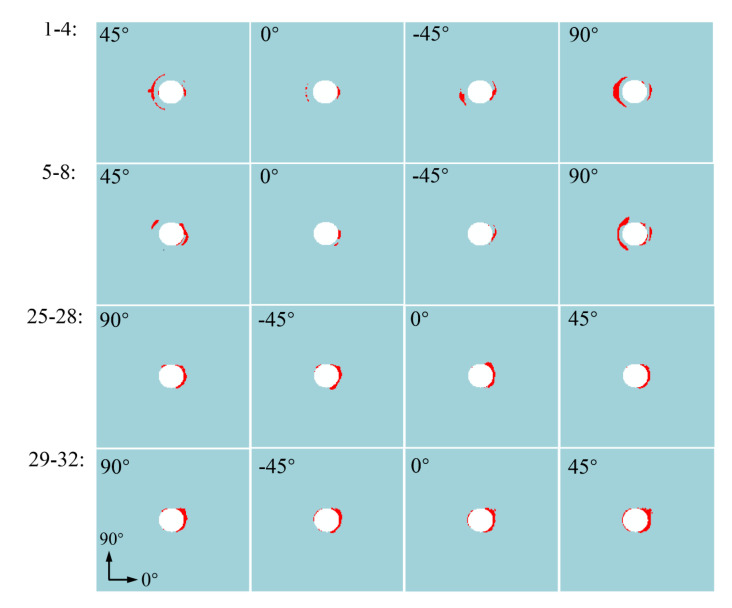
Thin-ply-laminated matrix damage state at point B.

**Figure 15:**
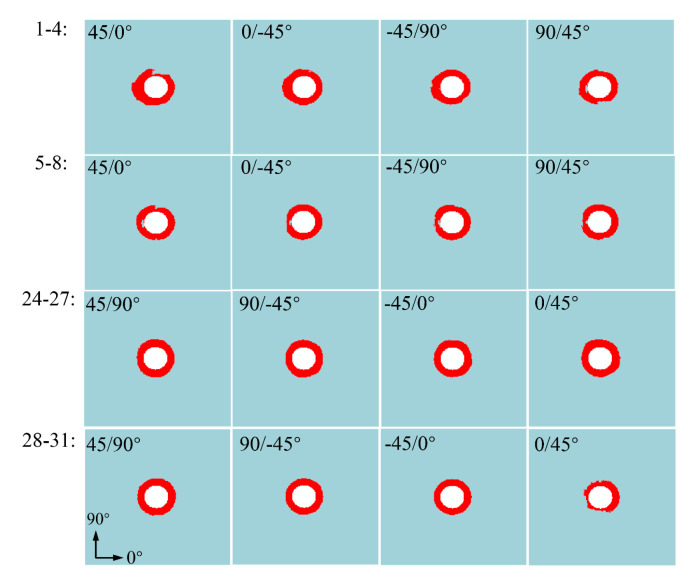
Cohesive element damage state of thin-ply-laminated at point B.

To further study the progressive damage mechanism of thin-ply composite structure, the status of in-plane fiber damage, in-plane matrix damage, and interlaminar cohesive element in the overlapping areas of the lap plate within eight layers up and down the surface is extracted respectively when the load peaked at point B of Figure 13. The layers were numbered 1–32 in sequence from top to bottom, and the interlayer cohesion elements were numbered 1–31 in sequence from top to bottom. The extraction results are shown in Figure 13, Figure 14 and Figure 15.

As shown in Figure 13, when the structure reaches the peak load-bearing capacity, the kinking failure commonly occurs in the fibers around the laminate hole, and it expands outward at a slow pace along the direction of the fiber. In the upper surface layer, the damage caused by fiber kinking occurs on a small scale in the 90° layer and the area of contact between the outermost layer and the nut. In the lower surface layer, this damage occurs on a small scale as well in all layers except the 90° layer. This also explains why the delamination damage occurring between the 90° fiber layers in the thin-ply laminate composite is limited, which is different from the extensive delamination damage caused in the extrusion process of conventional laminate. According to the state of matrix damage, as shown in Figure 14, the matrix damage occurring on the upper surface of the laminate concentrates in contact with the left edge of the nut, while the matrix damage on the lower surface concentrates in contact between the right side of the hole and the screw.

As confirmed by the distribution of matrix damage occurring around the hole of the laminate, the bolt deflects due to the secondary bending effect, which causes the left side of the nut to be embedded downwards and the right side to tilt upwards. Due to the embedding on the left of the nut and the increased extrusion of the screw in the middle, matrix damage occurs on the left of the upper surface and the right of the lower surface. According to the damage state on the right of the hole in Figure 14, the matrix damage on the right of the hole in the upper surface of several layers shows no outward expansion from the hole edge. Instead, it occurs before matrix damage occurs continuously at the hole edge. This is because the secondary bending effect leads to non-uniform extrusion of the screw on the laminate, and the shear effect caused by non-uniform extrusion leads to the shear failure of the matrix on the right of the hole before the occurrence of continuous matrix damage.

**Figure 16:**
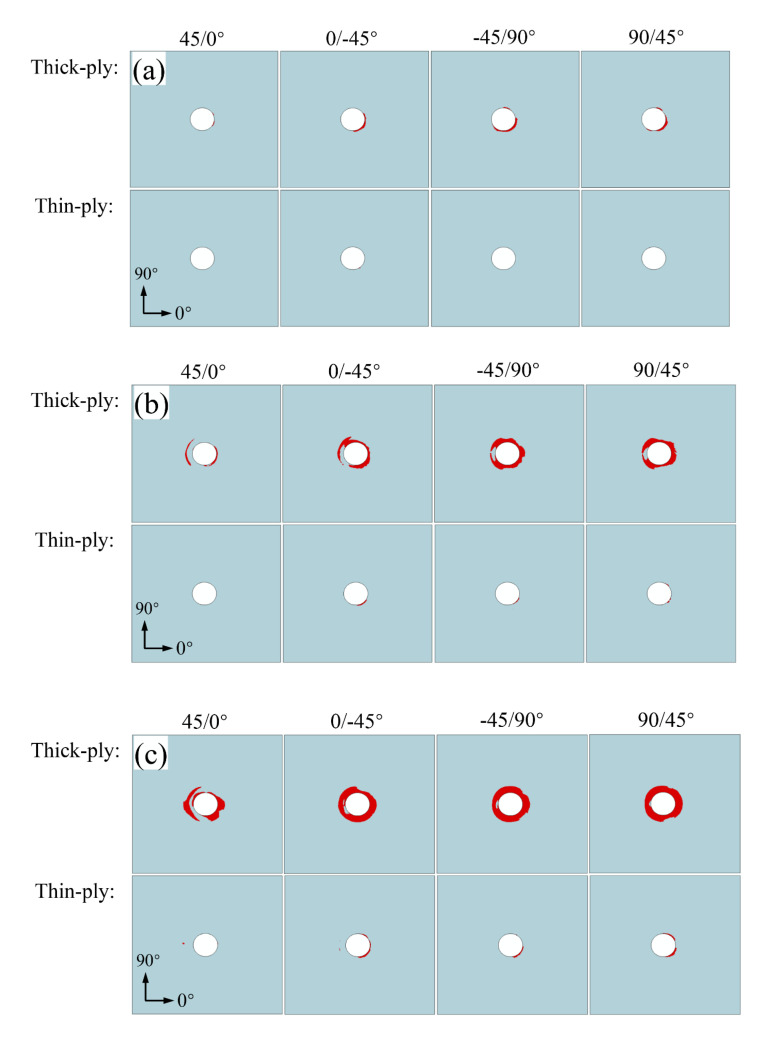
Delamination damage state of thick-ply-;laminated and thin-ply-laminated ((**a**) 300 MPa, (**b**) 400 MPa, and (**c**) 500 MPa).

According to the state of delamination damage around the hole of the laminate, as shown in Figure 15, when the single-lap structure of the thin-ply composite material reaches the peak load-bearing capacity, delamination damage occurs to a certain degree. To better characterize the damage initiation of the thin-layer composite on delamination expansion, the composite single-lap structure with a conventional thickness corresponding to the thin layer is built for comparison. The stacking sequence of composite single-lap structure with a conventional thickness is [45/0/–45/90]_2S_, and the total thickness is 2.08 mm, with 16 layers in total. The damage state of the cohesive element in the four layers on the upper surface of the lapping plate with a conventional thickness and the thin-ply one is respectively extracted when the stress reaches 300, 400, and 500 MPa, as shown in Figure 16.

As shown in Figure 16, no obvious delamination damage occurs on the upper surface of the four layers of the thin-ply single-lap structure at 300 MPa. However, delamination damage occurs to a certain degree on the upper surface of the four layers of the single-lap structure with a conventional thickness, especially in the region of early contact between the right side of the hole and the screw. When the load reaches 400 MPa, delamination damage occurs on a small scale in the thin-ply-laminated composite structure. At this time, the delamination damage caused to the conventional thickness materials expands from the area of contact on the right of the bolt to the surrounding area, and it also occurs around contact with the left edge of the nut. When the load reaches 500 MPa, delamination damage occurs extensively around the pore of the conventional thickness materials, but it occurs commonly around the pore of the thin-ply structure. By comparing the delamination damage states of thin-ply and thick-ply-laminated composite-bolted joint under the condition of 300 MPa and 500 MPa, the numerical simulation results indicate that the thin-ply composite initial delamination damage stress was about 60% higher than that of the conventional thick-ply. The simulation results are consistent with the experimental results of thin-ply-laminated composites and conventional thick-ply-laminated composites in Ref [13].

Huang et al. [7] proposed through the thin-ply experiment that by decreasing the ply thickness, higher interlaminar shear strength is obtained. According to the simulation results, the numerical model considering the in situ effect can well predict the interlayer enhancement effect. By comparing the development of delamination damage caused to the single-lap structures with a conventional thickness and thin-ply, the single-lap structures with thin-ply composite materials do not suffer extensive delamination damage that is common in the conventional thickness materials [32]. Ireman et al. [33,34,35] evidenced that the microstructure of thin-ply-laminated composite performs better in uniformity, which is more conducive to preventing intra-layer damage and the expansion of delamination damage between layers. In the paper, the numerical model of a thin-ply-laminated composite-bolted joint is created. At present, much research on thin-layer composites has been carried out, which contributes to further verification and improvement in the numerical model [4,36,37,38,39,40,41]. The single-lap structure of thin-laminated composite material will retain its overall bearing capacity when damage occurs in contact between the laminate hole and the bolt. Moreover, the stress in the damaged area will transfer to the surrounding area, thus forming a new stress riser and then enabling the structure to reach a new equilibrium. However, after damage occurs around the hole of the composite laminate with a conventional thickness, the continuous loading is quick to cause the overall failure of the structure, which loses the overall bearing capacity. The variation in this aspect between the thin material and the material with a conventional thickness provides a reference for the design of aviation structures in terms of safety.

## 4. Conclusions

The single-lap thin-ply-laminated composite-bolted joint under tension loading can exhibit various specific damage and failure modes of thin-ply composite structures, such as in situ effect, delamination inhibition, fiber compressive kinking failure, etc. As far as I know, there are merely related numerical simulation reports on bolted connections of thin-ply composites at present. A progressive damage model based on three-dimensional (3D) LaRC failure criterion combined with cohesive element is developed to study the damage evolution and failure mechanism of thin-ply-laminated composites jointed by a single-lap bolt.

It is shown that the predicted stress-displacement curves agree well with those of experimental observation. Our proposed model can not only capture some specific failure models, such as the fiber compressive kinking failure but also observe the whole interlaminar and intralaminar damage evolution, which is hard to characterize in real-time using an experimental tool. The predicted stress at the onset of delamination is 50% higher than that of the conventional thick materials, which is also consistent with experimental results. Moreover, the numerical model provides evidence that the microstructure of thin-ply-laminated composite performs better in uniformity, which is more conducive to inhibiting the intra-layer damage and the expansion of delamination damage between layers. This study on the damage inhibition mechanism of thin-ply provides a potential analytical tool for evaluating damage tolerance and bearing capabilities of thin-ply-laminated composite-bolted joints. 

## Figures and Tables

**Table 1:** Properties of CFRP single-lap material [25].

Material Type	Items	Value
T700/2510 solid element	Density (kg/m^3^)	*ρ* = 1520
	Modulus (GPa)	*E*_11_ = 127, *E*_22_ = *E*_33_ = 8.41, *G*_12_ = *G*_13_ = 4.21, *G*_23_ = 3.4
	Poisson’s ratio	*v*_12_ = *v*_13_ = 0.309, *v*_23_ = 0.3
	Strength (MPa)	*X*_T_ = 2200, *X*_C_ = 1470, *Y*_T_ = 49, *Y*_C_ = 199, S = 150
	Breaking energy (N/mm)	*G*_f_ = 133, *G*_kink_ = 40, *G*_IC_ = 0.227, *G*_IIC_ = 0.788
Cohesive element	Density (kg/m^3^)	*ρ*_1_ = 1520
	Modulus (N/mm^3^)	*K*_1_ = *K*_II_ = 6 × 10^4^
	Strength (MPa)	*N*_1_ = 38.5, *S*_1_ = *T*_1_ = 48.5
	Breaking energy (N/mm)	*G*_IC_ = 0.227, *G*_IIC_ = 0.788
TC21	Density (kg/m^3^)	*ρ*_2_ = 4510
	Modulus (GPa)	*E*_2_ = 110
	Strength (MPa)	*N*_2_ = 1100

**Table 2:** Expressions of in situ transverse tensile and shear strength [21].

Layer Type	Mathematical Expression
Embedded thick ply	$Y_{T}^{\mathrm{is}}=1.12\sqrt{2}Y_{T},\;S_{L}^{\mathrm{is}}=\sqrt{\frac{{\left(1+\beta \lambda G_{12}^{2}\right)}^{1/2}-1}{3\beta G_{12}}},\;\lambda =\frac{12{\left(S_{L}\right)}^{2}}{G_{12}}+18\beta {\left(S_{L}\right)}^{4}$
Embedded thin ply	$Y_{T}^{\mathrm{is}}=\sqrt{\frac{8G_{I}^{C}(L)}{\pi t\Lambda_{22}^{0}}},\;S_{L}^{\mathrm{is}}=\sqrt{\frac{{\left(1+\beta \lambda G_{12}^{2}\right)}^{1/2}-1}{3\beta G_{12}}},\;\lambda =\frac{48G_{\mathrm{II}}^{C}(L)}{\pi t}$
Outer surface thin ply	$Y_{T}^{\mathrm{is}}=1.79\sqrt{\frac{G_{I}^{C}(L)}{\pi t\Lambda_{22}^{0}}},\;S_{L}^{\mathrm{is}}=\sqrt{\frac{{\left(1+\beta \lambda G_{12}^{2}\right)}^{1/2}-1}{3\beta G_{12}}},\;\lambda =\frac{12{\left(S_{L}\right)}^{2}}{G_{12}}+18\beta {\left(S_{L}\right)}^{4}$
Outer surface thick ply	$Y_{T}^{\mathrm{is}}=Y_{T},\;S_{L}^{\mathrm{is}}=S_{L}^{}$

## Data Availability

The data presented in this study are available on request from the corresponding author.
